# Comparison of LFQ and IPTL for Protein Identification and Relative Quantification

**DOI:** 10.3390/molecules26175330

**Published:** 2021-09-02

**Authors:** Christina Johannsen, Christian J. Koehler, Bernd Thiede

**Affiliations:** Department of Biosciences, University of Oslo, 0316 Oslo, Norway; christina.johannsen@ibv.uio.no (C.J.); christian.koehler@ibv.uio.no (C.J.K.)

**Keywords:** IPTL, LFQ, quantitative proteomics, SILAC, TMT

## Abstract

(1) Background: Mass spectrometry-based quantitative proteome profiling is most commonly performed by label-free quantification (LFQ), stable isotopic labeling with amino acids in cell culture (SILAC), and reporter ion-based isobaric labeling methods (TMT and iTRAQ). Isobaric peptide termini labeling (IPTL) was described as an alternative to these methods and is based on crosswise labeling of both peptide termini and MS2 quantification. High quantification accuracy was assumed for IPTL because multiple quantification points are obtained per identified MS2 spectrum. A direct comparison of IPTL with other quantification methods has not been performed yet because IPTL commonly requires digestion with endoproteinase Lys-C. (2) Methods: To enable tryptic digestion of IPTL samples, a novel labeling for IPTL was developed that combines metabolic labeling (Arg-0/Lys-0 and Arg-d4/Lys-d4, respectively) with crosswise N-terminal dimethylation (d4 and d0, respectively). (3) Results: The comparison of IPTL with LFQ revealed significantly more protein identifications for LFQ above homology ion scores but not above identity ion scores. (4) Conclusions: The quantification accuracy was superior for LFQ despite the many quantification points obtained with IPTL.

## 1. Introduction

Liquid chromatography coupled to mass spectrometry (LC–MS) is the common tool for the analysis of proteins in complex mixtures due to major improvements in LC–MS during the last few decades [1]. MS-based proteomics facilitates rapid and sensitive data acquisition, which contributes to the improved identification and quantification of proteins and their posttranslational modifications (PTMs). Proteomics experiments commonly aim to determine differential abundances across proteins. In general, the relative quantification of protein abundances in complex mixtures can be achieved in two different ways: either by the incorporation of stable isotope labels or via label-free quantification (LFQ) [2].

LFQ is based on the comparison of intensities of precursor ions or spectral counts and has the advantage that labor-intensive and costly procedures of stable isotope labeling are not required. However, LFQ depends on very consistent sample preparation because the samples are processed independently and finally compared on the LC–MS data. Therefore, stable isotope labeling techniques are still widely used in quantitative proteomics because sample labeling and pooling are performed prior to LC–MS analysis. These methods can either be based on isotopic quantification and therefore rely on MS1 spectra or on MS2 spectra quantification using isobaric labeling. Most commonly, stable isotopic labeling is performed by metabolic labeling using stable isotope labeling with amino acids in cell culture (SILAC) [3], chemical stable isotopic dimethylation [4], and isobaric labeling using isobaric tagging for relative and absolute quantification (iTRAQ) [5], or tandem mass tags (TMT) [6]. SILAC requires the introduction of stable ^2^H, ^13^C, or ^15^N-enriched amino acids early in the experimental workflow during cell culture, which results in a robust and precise method enabling whole-proteome labeling and thus the quantification of living cells [3]. However, SILAC is difficult to apply on nondividing cells and human samples. A frequently used chemical derivatization for quantitative proteomics is isotopic labeling by dimethylation [4]. Reductive amination incorporates a dimethyl label at the N-terminal and *ε*-amino group of lysine in a fast and specific reaction. The dimethylation workflow only requires commercially available reagents at low cost [7]. Methods such as TMT or iTRAQ introduce isobaric labels that result in precursor ions of identical mass releasing reporter ions during fragmentation, which allow for quantification in MS2 spectra. Such methods enable the multiplexing of up to 18 samples, leading to their increasing popularity [8]. Even though multiplexing provides increased sample depth, it was shown to come with compromised quantitative accuracy in complex mixtures due to the co-isolation and co-fragmentation of interfering precursor ions [9]. While these effects can be prevented by, for example, additional MS3 scans, the additional steps either influence instrument duty cycles or need special equipment [10,11,12].

Another labeling technique called isobaric peptide termini labeling (IPTL) was introduced in 2009 [13]. For IPTL, peptides are crosswise isotopically labeled at the N- and C-terminus. The labeling reagents are chosen in isotopic variations so that the resulting mass of both labels is isobaric, but the individual label on each peptide terminus is different. Therefore, the quantitative difference of the peptide signal can be determined by the fragment ions of the corresponding MS2 spectra. The IPTL approach has been further developed using different labeling methods [14,15,16], triplex labeling [17,18], and customized software tools [19,20,21]. Moreover, pseudo-isobaric peptide termini labeling was developed, which is based on the co-fragmentation of isobaric isotopologues, which are molecular species of the same mass differing only in isotope composition [22,23]. This approach can be extended to 6-plex and 7-plex analyses [24,25]. Recently, collision-induced dissociation cleavable tags were developed to reduce MS2 spectrum complexity and to further improve the multiplexing capacity of IPTL [26].

Because of the multiple quantification points within MS2 spectra, it was assumed that IPTL can provide higher quantification accuracy than other quantification methods. However, a direct comparison of IPTL with other quantification techniques has not been performed yet because most of the IPTL approaches are based on protein digestion with endoproteinase Lys-C, thereby longer peptides are generated as with trypsin, which typically generates significantly less protein identifications [27,28]. Here, a novel approach for IPTL is presented, combining SILAC labeling with arginine-d4 and lysine-d4, tryptic digestion, and subsequent chemical labeling with N-terminal dimethylation. This approach allowed the comparison of the identification rates and quantification accuracy of LFQ and IPTL.

## 2. Results

### 2.1. Experimental Design and Effect of Labeling

**Figure 1:**
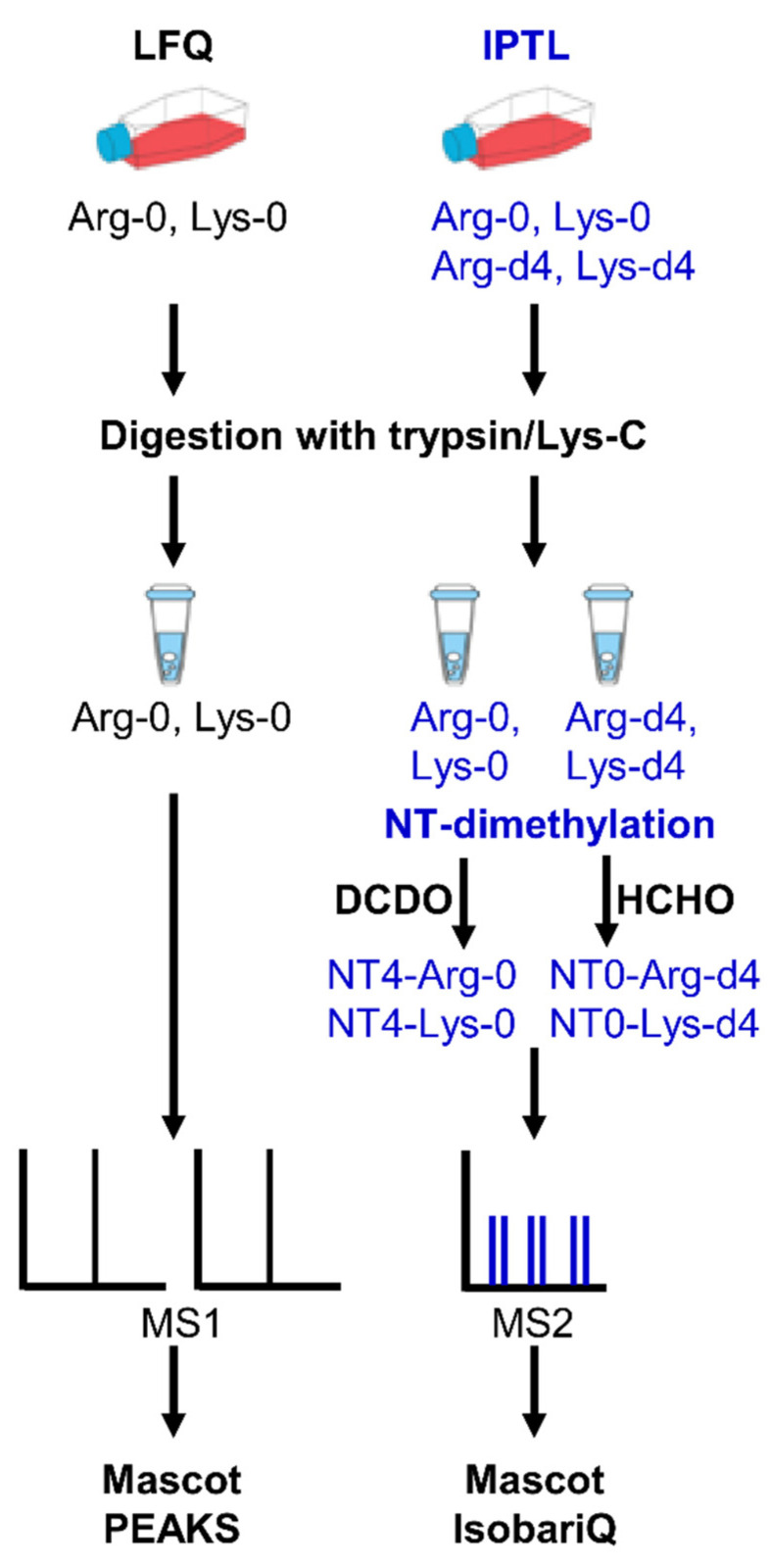
Workflow for the comparison of LFQ and IPTL.

The aim of this study was to compare the identification rate and quantification accuracy of IPTL with LFQ. A schematic overview of the experimental workflow is shown in Figure 1. The Jurkat T cell line was grown with arginine-0/lysine-0 for LFQ, and arginine-0/lysine-0 (light) and arginine-d4/lysine-d4 for IPTL, respectively. Metabolic labeling with arginine-d4 and lysine-d4 enabled the use of trypsin as an endoproteinase for IPTL. The IPTL samples were further crosswise labeled using N-terminal dimethylation to obtain isobaric peptides. In total, eight replicates of LFQ and IPTL were analyzed by LC–MS and quantified on the MS1 (LFQ) or MS2 (IPTL) level, respectively (Figure 1). Mascot 2.4 and PEAKS Studio X were used for protein identification. For relative quantification, PEAKS Studio X was applied to LFQ data. Because PEAKS Studio cannot be applied to quantify IPTL data, these data were quantified with IsobariQ.

Jurkat T cells were cultured in different media with Arg/Lys or Arg-d4/Lys-d4. The proteins were digested with trypsin/Lys-C. For IPTL, Arg/Lys and Arg-d4/Lys-d4 labeled proteins, respectively, were subsequently crosswise modified by N-terminal (NT) dimethylation using formaldehyde-d2 (DCHO) or formaldehyde (HCHO) to obtain isobaric peptides. After LC–MS analysis, the data were analyzed using Mascot, PEAKS, and IsobariQ.

### 2.2. Comparison of Protein Identification of LFQ and IPTL

In general, the experiments showed that the number of acquired MS2 spectra was slightly higher for LFQ (655,323) than for IPTL (633,111) (Table 1). Because PEAKS X can identify chimera MS2 spectra, we expected that many would be found using IPTL; however, more chimera MS2 spectra were found for LFQ (375,534 vs. 272,940) (Table 1). In addition, LFQ resulted in significantly more PSMs than IPTL (PEAKS 431,057 vs. 237,423; Mascot 347,660 vs. 131,557 above identity threshold and 357,157 vs. 126,408 above homology threshold), which is also reflected by the percentage of PSMs to MS2 scans (PEAKS 66% vs. 43%; Mascot 53/55% vs. 21/20%) (Table 1). This might be due to the much higher ion score threshold of 31 and 38 for IPTL in comparison to 22 and 25 for LFQ. However, LFQ revealed more protein groups than IPTL above the homology threshold but not above the identity threshold (4797 vs. 5215).

**Figure 2:**
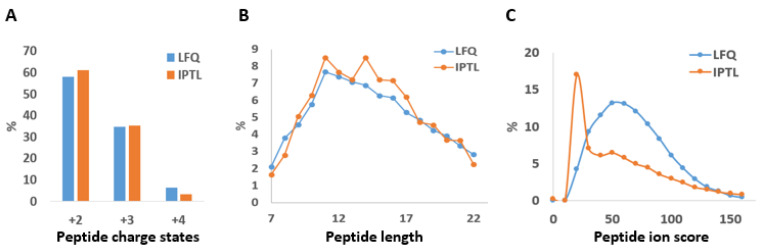
Characteristics of quantified peptides using LFQ and IPTL. (**A**) The peptide counts with charges of 2+ to 4+ is shown, (**B**) the peptide length are compared, and (**C**) the distribution of the peptide ion scores from Mascot is displayed.

To explain the differences in peptide identifications, physicochemical properties such as peptide charge and length were compared but revealed no striking differences (Figure 2A,B). However, the peptide scores were lower in IPTL compared to LFQ (Figure 2C).

### 2.3. Comparison of Protein Quantification of LFQ and IPTL

Four against four replicates were compared using PEAKS for protein quantification of the LFQ data.

Because PEAKS is not applicable for IPTL data, Mascot dat files were quantified using IsobariQ for IPTL. The number of quantified protein groups was significantly higher with LFQ (3040) compared to IPTL (2021) (Table 2). Approximately the same percentage of quantified vs. identified was obtained. LFQ revealed six protein groups with a fold change of 1.5–2 and only a single protein above 2, showing the high reliability of LFQ for quantification. In contrast, a significant number (78) of protein groups with a fold change between 1.5 and 2 and 16 protein groups above a fold change of 2 were found with IPTL. Considering the fact that 1475 of the protein groups were quantified in both datasets, it can be concluded that overall the quantification accuracy is higher using LFQ with PEAKS than using IPTL with IsobariQ. It must be pointed out that at least two peptides were applied in PEAKS for LFQ data analysis, whereas the minimal number of quantified peptides (PSMs) per protein was set to four in IsobariQ for IPTL. It is not possible to set the number of unique peptides in IsobariQ, but such a setting might improve the quantification accuracy for IPTL data.

## 3. Discussion

A direct comparison of LFQ against IPTL was performed. For this purpose, it was necessary to establish a novel workflow for the analysis of tryptic peptides using IPTL. Although the number of acquired MS2 spectra was quite similar, the number of PSMs for IPTL was around 50% less using PEAKS and more than 60% less using Mascot in comparison to PSMs from LFQ. A reason for the reduced identification rate is the much higher MS2 ion score threshold for IPTL. In fact, four additional variable modifications were used for IPTL database searches, which increased the search space. Yet, it was surprising that the number of identified proteins above the identity ion score threshold was even higher with IPTL than with LFQ. In contrast, the opposite was observed above the homology ion score threshold. The difference between the identity and homology ion scores was three for LFQ, whereas it was seven for IPTL. In general, Mascot ion scores above 30 are highly reliable and excluding MS2 spectra with even higher ion scores is certainly a reason for the lower PSM rate. Furthermore, we were surprised that PEAKS recognized less chimera spectra in IPTL than LFQ. Considering the concept of IPTL, all MS2 spectra in a 1:1 mixture should contain chimera spectra, i.e., the software should find one IPTL labeled version in the first-pass MS2 database search (e.g., NT0-CT4), and the other (e.g., NT4-CT0) in a second-pass MS database search. It is unclear why these chimera MS spectra were not found by PEAKS. Actually, IPTL MS2 spectra contain more fragment information about the corresponding peptides than LFQ. Thus, a dedicated protein identification software fully exploiting IPTL data is required to take full advantage of the approach and might even lead to higher numbers of protein identifications.

Several quantification points per peptide with reciprocal ratios for N-terminal and C-terminal fragments are obtained with IPTL. Thus, statistical analysis of the data for each peptide can be performed. It was proposed that this feature could lead to high quantification accuracy. However, the result of this study showed that the multiple quantification points which are contained in the MS spectra in IPTL did not result in any higher quantification accuracy than LFQ.

## 4. Materials and Methods

### 4.1. Cell Culture and Incorporation of Stable Isotope Labeling by Amino Acids in Cell Culture (SILAC)

If not stated differently, all chemicals were purchased from Merck Life Science AS/Sigma Aldrich, Oslo, Norway AS. The Jurkat T-cell line E6 was maintained in RPMI tissue culture medium (Gibco BRL, Karlsruhe, Germany) supplemented with 10% fetal calf serum and 200 mg/L proline and maintained in a humid incubator at 37 °C and 5% CO_2_ to reach a density of 1 × 10^6^ cells/mL. The cell culture was split into three culturing bottles from which two were cultured in normal RPMI-1640 media for controls containing Lys-0 (L-lysine [^12^C_6_, ^14^N_2_]) and Arg-0 (L-arginine [^12^C_6_, ^14^N_4_]), and one was cultured in SILAC media (Thermo Scientific, Oslo, Norway) containing Lys-d4 (L-lysine [^2^H_4_]) and Arg-d4 (L-arginine [^2^H_4_]) (Eurisotop, Saint Aubin, France). Cells were harvested after reaching a density of 1 × 10^6^ cells/mL, resuspended in 1 mL PBS, and centrifuged at 200 *g*. Cell pellets were frozen at −80 °C.

### 4.2. Cell Lysis, Protein Precipitation, and in-Solution Trypsin/LysC Digestion

Cell pellets were thawed and 800 µL cell lysis buffer (Invitrogen, Oslo, Norway) was added, followed by homogenization and mechanical breakage with a blue pestle. The samples were centrifuged at 16,000× *g* for 20 min at 4 °C in a Heraeus Biofuge pico (Kendro, Hanau, Germany) and the supernatant was divided in 20 μL aliquots. An aliquot was precipitated with 80 µL 100% ice-cold acetone over night at −20 °C. After centrifugation at 16,000× *g* for 20 min at 4 °C, the supernatant was discarded, and the precipitate was washed three times with acetone. The pellet was dissolved in 50 µL 6 M urea, and DTT was added to a final concentration of 10 mM, mixed thoroughly and incubated at 37 °C for 30 min using a Thermomixer (Eppendorf, Hamburg, Germany). The reduction was followed by alkylation using iodoacetamide to a final concentration of 25 mM, incubated for 60 min in the dark at room temperature. Excess alkylation reagent was quenched by adding DTT to a final concentration of 30 mM and left for 30 min at 37 °C. For digestion, 240 µL 50 mM ammonium bicarbonate buffer and Trypsin/LysC (Promega, Madison, WI, USA) with an enzyme to protein ratio of 1:200 were added and incubated for 18 h at 37 °C. The digestion was stopped by adding formic acid to a final concentration of 1%. The tryptic peptides were purified by solid phase extraction using Strata C18-E SPE cartridge (Phenomenex, Værløse, Denmark) and evaporated to dryness using a Speed Vac concentrator (Eppendorf, Hamburg, Germany). The Strata C18-E SPE cartridges were activated using 500 µL acetonitrile and equilibrated with three times 500 µL 1% formic acid in water. The sample was loaded by passing through the column material three times. The column was washed using 500 µL water and the peptides were eluted with 500 µL 50% acetonitrile.

### 4.3. N-Terminal Dimethylation for IPTL Samples

The frozen, dried, and purified tryptic digest was thawed and dissolved in 40 µL 1% acetic acid, pH 2.7, and vortexed for about 5 min. A total of 2 µL 4% formaldehyde or formaldehyde-d2 in water and 2 µL sodium cyanoborohydride were added to a final concentration of 30 mM, mixed thoroughly, and left for incubation for 30 min. Subsequently, 8 µL of 1% ammonia hydroxide was added, vortexed, and incubated for 1 min. The reaction was stopped by adding 1 µL 5% formic acid. The tryptic peptides were purified with 10 µL OMIX-C18 Tips and dried using a Speed Vac concentrator. The OMIX-C18 Tips were activated using 50 µL acetonitrile and equilibrated with three times 50 µL 1% formic acid in water. The sample was loaded by passing through the column material three times. The column was washed using 50 µL of water and eluted with 10 µL 70% acetonitrile.

### 4.4. LC–MS Analysis

For LC–MS/MS analysis of the Jurkat T cell proteome, the dried peptides were dissolved in 10 µL 1% formic acid in water. For LFQ, 10 µL of the control was taken to be measured in eight replicates. For IPTL, 5 µL of control (Arg-0, Lys-0) and 5 µL of SILAC sample (Arg-d4, Lys-d4) were crosswise N-terminally dimethylated (d4 and d0, respectively), and the isobaric peptides were mixed and measured in eight replicates. The samples were injected into a Dionex Ultimate 3000 nano-UHPLC system (Sunnyvale, CA, USA) connected to a quadrupole-Orbitrap (QExactive) mass spectrometer (Thermo, Bremen, Germany). For ionization, a nano electrospray ion source was used and the UHPLC was equipped with an Acclaim PepMap 100 column (C18, 2 µm beads, 100 Å, 75 μm inner diameter, 50 cm length) (Dionex, Sunnyvale CA, USA). The flow rate was 0.3 µL/min with a solvent gradient of 4–35% B in 180 min. Solvent A was 0.1% formic acid and solvent B was 0.1% formic acid/90% acetonitrile. The mass spectrometer was operated in data-dependent acquisition and switched automatically between MS1 and MS2 acquisition. Survey full-scan MS1 spectra (from *m*/*z* 400 to 2000) were acquired in the Orbitrap with a resolution of 70,000 at *m*/*z* 200 (after accumulation to a target of 1,000,000 ions in the C-trap). Top ten most intense ions were isolated and fragmented in the HCD cell using high-energy collision dissociation (HCD) and a target value of 1 × 10^5^ and an injection time of 100 ms. The mass spectrometer was operated in the data-dependent mode to automatically switch between MS1 and MS2 acquisition. Target ions already selected for MS2 were dynamically excluded for 15 s. General mass spectrometry conditions were as follows: electrospray voltage, 2.0 kV; no sheath and auxiliary gas flow; heated capillary temperature of 275 °C; normalized HCD collision energy 28%. Ion selection threshold was set to 10,000 counts, and an isolation width of 2.0 Da was used.

### 4.5. Data Analysis

For further analysis, the raw files were converted to Mascot generic format (.mgf) files using ProteoWizard Analysis 3.0.331 applying peak picking: 2, filter: MS2 deisotope, filter: MS2 denoise 6, 100. For protein identifications, PEAKS Studio X+ and Mascot 2.4 were used. For relative quantification, LFQ samples were quantified using PEAKS Studio X+, whereas Mascot 2.4 in combination with IsobariQ version 2.0a was applied to IPTL data [19]. General search parameters were as follows: search against Swiss-Prot database (human, 20,431 sequences), trypsin was selected as enzyme with one allowed missed cleavage site, precursor ion tolerance of 10 ppm, and MS2 fragment tolerance of 0.05 Da. Carbamidomethylation of cysteines was set as fixed modification and N-terminal protein acetylation and methionine oxidations as variable modifications. For IPTL, Lys-d4, Arg-d4, dimethylation-d0, and dimethylation-d4 were chosen as variable modifications with satellite ions [29]. False discovery rates were calculated in Mascot using automatic decoy database searches [30] and in PEAKS using a decoy fusion method [31]. The mass spectrometry proteomics data have been deposited in the ProteomeXchange Consortium via the PRIDE partner repository [32] with the dataset identifier PXD026942.

For quantification of LFQ data using PEAKS, the following parameters were applied: quality ≥ 4, average area ≥ 1 × 10^−5^, charge: 2–5, peptide ID count per group ≥ 1 in at least 2 samples per group, significance ≥ 0, and significance method: ANOVA with at least 2 peptides. Ten internal standard proteins were used for normalization. For quantification of IPTL data using IsobariQ, the Mascot dat file was used with the following parameters: ignore peptide assignments with ion score below 20, peptide scoring standard for identification and minimal number of quantified peptides (PSMs) per protein: 4, require all labeling schemas, require bold red, and use unique and razor peptide for quantification. Normalization in IsobariQ was performed by division by median in utilizing the ratios of all peptides (reporter ions) or quant points.

## Figures and Tables

**Table 1:** Protein identification for LFQ and IPTL. Eight replicates for LFQ and IPTL were analyzed by LC–MS and proteins identified with PEAKS X and Mascot 2.4. The numbers of MS2 scans and peptide–spectrum matches (PSMs) are displayed. The number of chimera spectra is reported in PEAKS, and the identity and homology threshold is provided by Mascot 2.4.

Software	Approach	M2 Scans (Chimera)	PSMs	PSMs/MS2 Scans	Identity (I) and Homology (H) Ion Score Threshold	Protein Groups
PEAKS X	LFQ	655,323 (375,534)	431,057	66%	-	5317
PEAKS X	IPTL	633,111 (272,940)	237,423	43%	-	4355
Mascot 2.4	LFQ	655,323	347,660 357,157	53%	I: 22 H: 25	4797 4603
Mascot 2.4	IPTL	633,111	131,557 126,408	21% 20%	I: 31 H: 38	5215 3607

**Table 2:** Protein quantification for LFQ and IPTL. Eight replicates for IPTL and four against four replicates for LFQ were compared. Quantitative data analysis was performed with PEAKS X for LFQ and with Mascot and IsobariQ for IPTL. For IPTL, protein groups above the homology ion score are displayed.

Software	Approach	Protein Groups	Quantified Proteins	Quantified/ Identified Proteins (%)	Fold Change 1–1.5	Fold Change 1.5–2	Fold Change >2
PEAKS X	LFQ	5317	3040	57%	3033	6	1
Mascot 2.4/ IsobariQ	IPTL	3607	2021	56%	1927	78	16

## Data Availability

The mass spectrometry proteomics data have been deposited in the ProteomeXchange Consortium via the PRIDE partner repository [32] with the dataset identifier PXD026942.

## Sample Availability

Samples of the compounds are not available from the authors.
